# Aberrations of TACC1 and TACC3 are associated with ovarian cancer

**DOI:** 10.1186/1472-6874-5-8

**Published:** 2005-05-26

**Authors:** Brenda Lauffart, Mary M Vaughan, Roger Eddy, David Chervinsky, Richard A DiCioccio, Jennifer D Black, Ivan H Still

**Affiliations:** 1 Department of Cancer Genetics, Roswell Park Cancer Institute, Elm and Carlton Streets, Buffalo, New York, 14263, USA; 2 Department of Pharmacology and Therapeutics, Roswell Park Cancer Institute, Elm and Carlton Streets, Buffalo, New York, 14263, USA; 3 Gilda Radner Familial Ovarian Cancer Registry, Roswell Park Cancer Institute, Elm and Carlton Streets, Buffalo, New York, 14263, USA

## Abstract

**Background:**

Dysregulation of the human Transforming Acidic Coiled Coil (TACC) genes is thought to be important in the development and progression of multiple myeloma, breast and gastric cancer. Recent, large-scale genomic analysis and Serial Analysis of Gene Expression data suggest that TACC1 and TACC3 may also be involved in the etiology of ovarian tumors from both familial and sporadic cases. Therefore, the aim of this study was to determine the occurrence of alterations of these TACCs in ovarian cancer.

**Methods:**

Detection and scoring of TACC1 and TACC3 expression was performed by immunohistochemical analysis of the T-BO-1 tissue/tumor microarray slide from the Cooperative Human Tissue Network, Tissue Array Research Program (TARP) of the National Cancer Institute, National Institutes of Health, Bethesda, MD, USA. Tumors were categorized as either positive (greater than 10% of cells staining) or negative. Statistical analysis was performed using Fisher's exact test and p < 0.05 (single comparisons), and p < 0.02 (multiple comparisons) were considered to be significant. Transgenomics WAVE high performance liquid chromatography (dHPLC) was used to pre-screen the TACC3 gene in constitutional DNA from ovarian cancer patients and their unaffected relatives from 76 families from the Gilda Radner Familial Ovarian Cancer Registry. All variant patterns were then sequenced.

**Results:**

This study demonstrated absence of at least one or both TACC proteins in 78.5% (51/65) of ovarian tumors tested, with TACC3 loss observed in 67.7% of tumors. The distribution pattern of expression of the two TACC proteins was different, with TACC3 loss being more common in serous papillary carcinoma compared with clear cell carcinomas, while TACC1 staining was less frequent in endometroid than in serous papillary tumor cores. In addition, we identified two constitutional mutations in the TACC3 gene in patients with ovarian cancer from the Gilda Radner Familial Ovarian Cancer Registry. These patients had previously tested negative for mutations in known ovarian cancer predisposing genes.

**Conclusion:**

When combined, our data suggest that aberrations of TACC genes, and TACC3 in particular, underlie a significant proportion of ovarian cancers. Thus, TACC3 could be a hitherto unknown endogenous factor that contributes to ovarian tumorigenesis.

## Background

It is apparent that for a normal cell to develop into a highly delocalized metastatic cancer, multiple genetic events are required to overcome the normal mechanisms that control the growth and development of healthy tissue. About 10% of ovarian cancer patients inherit a familial predisposition, and of those cases, only 35–50% can be attributed to the inheritance of defects in the BRCA1 and BRCA2 tumor suppressor genes [1,2]. In addition, BRCA1 and BRCA2 mutations are not directly involved in the initiation events leading to the development of sporadic tumors, indicating that additional, as yet unidentified genes must play a significant role in the etiology of both familial and sporadic ovarian cancer. In ovarian cancer, comparative genomic hybridization (CGH), multicolor spectral karyotyping (SKY), and loss of heterozygosity (LOH) studies have identified several regions of the genome that may contain novel genes involved in the development and progression of ovarian cancer [3-5]. These techniques have indicated that deletions or rearrangements of 4p16 and 8p11, the loci for TACC3 and TACC1 respectively, commonly occur in 40% of ovarian cancer cell lines and primary tumors from both familial and sporadic cases [3-5]. SAGE (Serial Analysis of Gene expression) analysis further suggests that TACC3 and TACC1 are downregulated in ovarian tumors and ovarian cancer cell lines [6]. Thus, based upon both the location of TACC1 and TACC3 in regions consistently associated with ovarian cancer [3,5] and SAGE expression data [6], we have set out to determine the occurrence of alterations of these TACCs in ovarian cancer.

## Methods

### Serial Analysis of Gene Expression (SAGE)

The results of SAGE analysis of libraries generated by the method of Velculescu et al [7] were downloaded from the SAGEMAP section of the **G**ene **E**xpression **O**mnibus website at the National Center for Biotechnology Information [6], and critically assessed for reliability to specifically predict expression of TACC3 and TACC1 in ovarian tissue and tumors. SAGE profiles used for TACC3 and TACC1 were [8] and [9], respectively.

### Tissue and tumor microarrays

T-BO-1 and IMH-343 tissue and tumor microarray slides were obtained from the Cooperative Human Tissue Network, Tissue Array Research Program (TARP) of the National Cancer Institute, National Institutes of Health (Bethesda, MD, USA) and Imgenex Corporation (San Diego, CA, USA), respectively. Each of these microarrays contain normal tissues that are known to express TACC1 and TACC3 [10-12]. Clear cell carcinomas on the tissue/tumor slides, which cannot be graded using the World Health Organisation or FIGO systems [13], were classified as grade 3, as recommended by the NCCN practice guidelines [14].

### Immunohistochemical staining

Immunohistochemical procedures were first optimized using formalin-fixed, paraffin-embedded MCF7 and HT-29 cell lines prepared in our laboratory. Conditions were then further optimized using mixed normal/tumor human tissue microarrays (Imgenex Corporation).

Tumor microarrays were deparaffinized in three changes of xylene and rehydrated using graded alcohols at room temperature. Endogenous peroxidase was quenched with aqueous 3% H_2_O_2 _for 20 min. and washed with PBS-Tween20 (PBS/T). Antigen retrieval was performed with citrate buffer pH 6.0 by heating twice in a microwave for 10 min., and then cooling to room temperature for 15 min. Following a PBS/T wash, the slides were placed in a humidity chamber and 0.03% w/v casein in PBS/T was applied to the tissue sections for 30 min. to block non-specific binding. This was then replaced with the primary anti-TACC3 antibody (#sc5885, Santa Cruz Biotech., Santa Cruz, CA, USA) at 1 μg/ml and left overnight at 4°C. As a negative control, a duplicate slide was incubated with antibody that had been precompeted with 10-fold excess of TACC3 peptide competitor [Santa Cruz #sc5885P] for 2 hr. at room temperature prior to use. The slides were then washed with PBS/T, followed by the biotinylated secondary antibody [Santa Cruz #sc2042 Donkey anti-goat] for 30 min. A PBS/T wash was followed by incubation with streptavidin-peroxidase reagent [#50-242, Zymed, Carlsbad, CA, USA] for 30 min. PBS/T was used as a wash and then chromogen DAB [#K3466, DAKO, Carpinteria, CA, USA] was applied for 5 min. (color reaction product – brown). The slides were then counterstained with Hematoxylin (blue in figures), dehydrated, cleared and finally coverslipped.

Antigen retrieval for TACC1 was essentially as described above. Slides were then incubated overnight at 4°C with primary anti-TACC1 antibody (#07-229, Upstate, Lake Placid, NY, USA) at 1 μg/ml. Concentration matched rabbit IgG (1 μg/ml) was used on a duplicate slide, instead of the primary TACC1 antibody, as a negative control for immunostaining. The slides were then placed on a DAKO autostainer with the following program; 1) one PBS/T wash; 2) incubation with a biotinylated secondary antibody [Vector Elite kit] for 30 min.; 3) one PBS/T wash; and then 4) incubation with ABC reagent [Vector Elite kit] for 30 min. PBS/T was used as a final wash and chromogen-DAB [DAKO] was applied for 5 min. (color reaction product – brown). The slides were then counterstained with Hematoxylin and dehydrated, cleared and coverslipped.

### Image analysis

Tumor microarrays were examined by light microscopy and cores were categorized into either positive (+) or negative (-) staining for TACC3 and TACC1, with (+) staining representing those cores where greater than 10% of the cells had detectable staining, otherwise they were scored (-). No staining was observed in the negative controls, which were serial tumor microarray slides that were incubated with either peptide block or IgG depending upon the test antibody. Positive controls were normal tissues on each tissue and tumor array, and normal ovarian surface epithelium. Images were captured using a Nikon eclipse E600 microscope with Spot insight digital camera and Spot Advanced software version 4.0.1 (Diagnostic Instruments, MI, USA).

### Statistical analysis

Fisher's exact test was performed using GraphPad Prism Version 3.03, (GraphPad Software, San Diego, California, USA, ). All tests were two-sided and p < 0.05 and p < 0.02 were considered to be significant for single and multiple comparisons respectively.

### Mutation analysis using Transgenomic WAVE dHPLC

The TACC3 gene was screened for mutations using the Transgenomic WAVE dHPLC system [15]. The sequence of each fragment to be amplified by the polymerase chain reaction (PCR) was first analyzed using the WAVE melting profile software program to determine gradient and column temperature conditions for each PCR product. Individual exons and intronic regions encompassing the 5' and 3' splice sites were then amplified from a standard commercial normal human placental DNA sample (conditions and primer sequences available on request). These PCR products were then used to establish the actual melting profiles for each exon. Test samples were mixed with the standard normal control PCR product at a 1:1 ratio and denatured at 95°C for 5 min., followed by slow cooling to room temperature at the rate of 0.1°C every four seconds to enhance heteroduplex formation. WAVE dHPLC analysis was then performed and patterns compared with matched normal sibling controls (non-symptomatic) and the unmixed normal control pattern. For all variant patterns detected, aliquots from the original PCR reaction were then directly sequenced by the Roswell Park Cancer Institute Biopolymer core facility. Variant sequences were cross-referenced against GenBANK and Single Nucleotide Polymorphism databases [16], to test for occurrence in a wider population sample. New gene alterations that were found only in cDNAs from normal tissues in the databases, or found in normal and affected individuals in our test subjects were labelled as polymorphisms.

All investigations were performed after approval by the Roswell Park Cancer Institute institutional review board.

## Results

### Expression analysis of TACC1 and TACC3 in ovarian tumors

**Figure 1:**
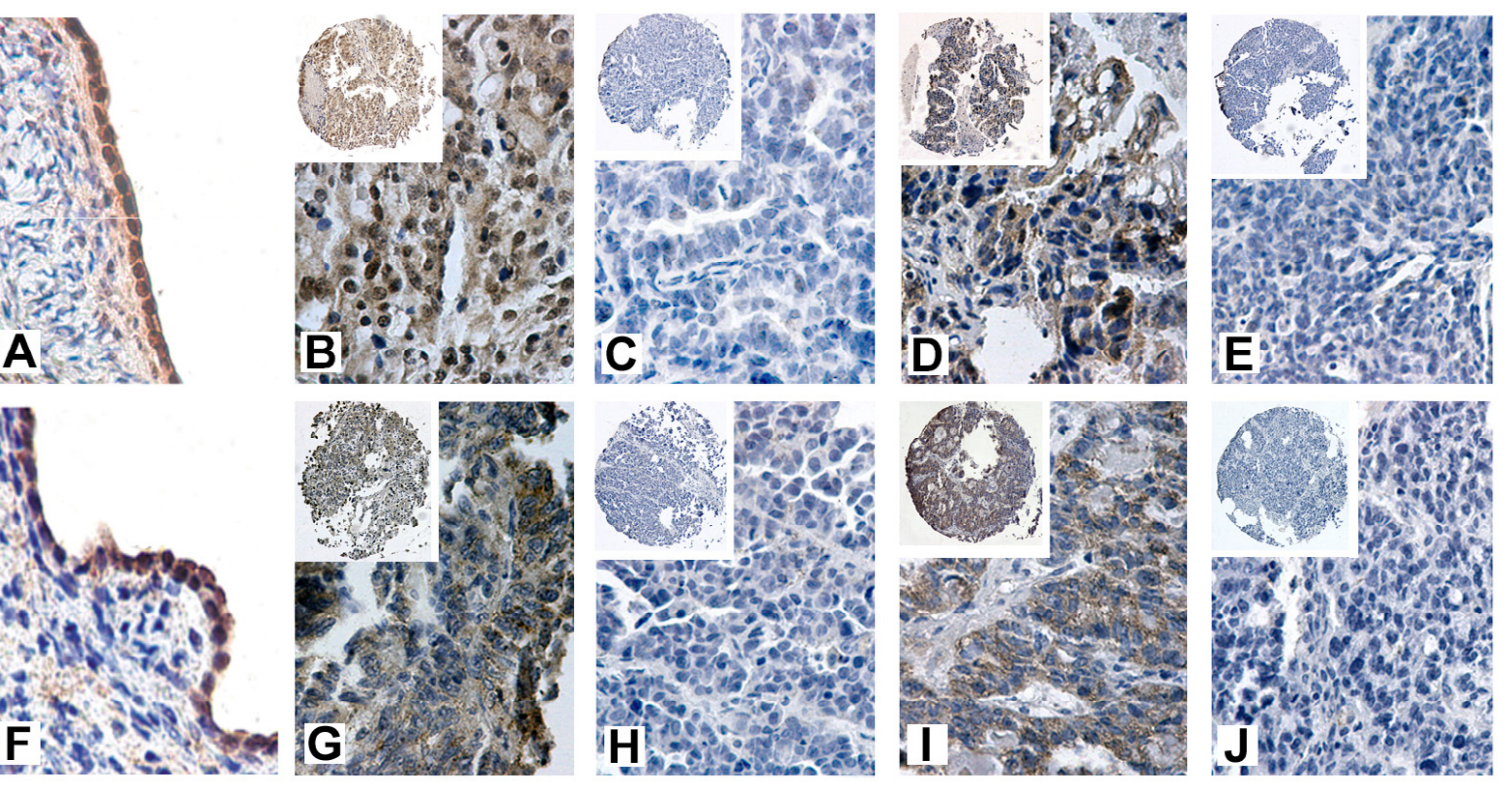
**Immunohistochemical analysis of tumor micoarrays**. Representative normal human ovarian surface epithelium and tumor cores stained for TACC1 (panels A-E), and TACC3 (panels F-J) proteins to show positive and negative staining. TACC protein expression is detected as a brown signal against the blue Hematoxylin counterstain. In all cases where TACC3 expression was observed, the protein was excluded from the nucleus of the ovarian tumor cells, unlike the observable nuclear and cytoplasmic expression of TACC1. A, normal ovarian surface epithelium with nuclear/cytoplasmic TACC1 staining; B, serous papillary TACC1 +ve; C, serous papillary TACC1 -ve; D, endometroid TACC1 +ve; E, endometroid TACC1 -ve; F, normal ovarian surface epithelium with nuclear TACC3 staining; G, serous papillary TACC3 +ve; H, serous papillary TACC3 -ve; I, clear cell TACC3 +ve; J, clear cell TACC3 -ve. A-H: Main panel original magnification ×40; insets show whole tumor core at original magnification ×10.

**Figure 2:**
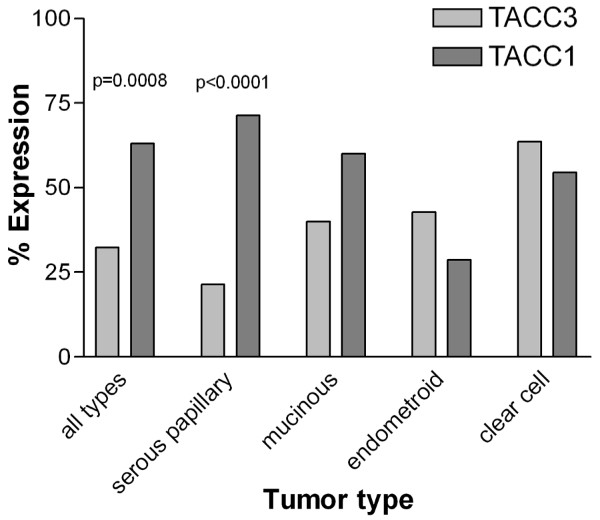
**Summary chart of the expression of TACC1 and TACC3 relative to ovarian tumor type**. The difference between expression of TACC3 and TACC1 in all types of tumors, and the serous papillary subtype in particular, was significant (Fisher's exact test, p = 0.0008, and p < 0.0001 respectively).

**Figure 3:**
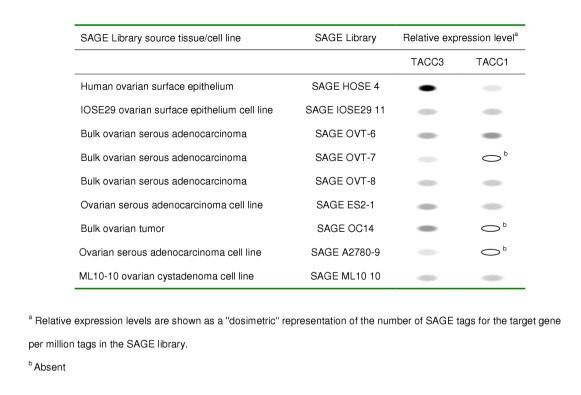
**Serial Analysis of Gene Expression for TACC1 and TACC3 in ovarian cancer**.

TACC1 and TACC3 are expressed in the epithelia of a number of different tissues, including the mammary gland and the ovary [17-19] (Fig. 1), suggesting that these proteins may be required for the normal maintenance of the epithelial component of organs within the body. Published SAGE (Serial Analysis of Gene Expression) analysis suggests that TACC1 expression is absent in 3 out of 7 bulk ovarian tumors and cell lines (Figure 3). Furthermore, TACC3 is downregulated in all 7 of the bulk ovarian tumors and tumor cell lines, compared with the normal human ovarian surface epithelium (HOSE) (Figure 3) [6]. Notably, similar to the two transciption factors GATA4 and GATA6 [20], TACC3 downregulation occurs in IOSE29, an immortalized ovarian surface epithelial cell line, suggesting that loss of TACC3 expression may be an early event during the immortalization process. To determine whether the pattern of gene expression noted by SAGE analysis is also evident in patient tumors, we assessed ovarian tumor microarrays from the National Cancer Institute multi-tumor tissue microarray (TARP) facility for expression of both proteins. 65 of the 75 OSE tumors on the tissue/tumor microarray could be evaluated for both TACC1 and TACC3 expression after the immunostaining procedure. Representative images demonstrating positive or negative tumor staining compared to normal human ovarian surface epithelium are shown in Fig. 1. A total of 21 (32.3%) and 41 (63.1%) showed positive staining for TACC3 and TACC1 respectively (Table 1). Thus, 44 (67.7%) and 24 (36.9%) could be categorized as absent or minimal expression for TACC3 and TACC1 respectively, similar to the results observed in the SAGE analysis. In addition, the allocation of TACC1 and TACC3 status to each core showed that 78.5% of the tumors exhibited negative immunoreactivity for one or both proteins. Within the ovarian tumor set tested (n = 65), there was a significant difference in the expression of TACC1 compared to TACC3 (Fisher's exact test, p = 0.0008) (Fig. 2). Expression of neither protein was associated with tumor grade. In all cases where TACC3 expression was observed, the protein was excluded from the nucleus (Fig. 1), contrary to the predominantly nuclear localization observed in the normal ovarian surface epithelium *in vivo *[17-19] (Fig 1). These results indicate that tumors derived from the ovarian surface epithelium exhibit low/negative expression or aberrant localization of TACC3, which may biologically contribute to development of these tumors.

**Table 1:** Distribution of TACC1 and TACC3 expression in ovarian tumors

Tumor type	**TACC3^a^**				**TACC1^a^**				**TACC3 & TACC1^b^**
	
	Tumor grades^c^				Tumor grades^c^				Tumor grades^c^
	I	II	III	all	I	II	III	all	all
serous papillary	1^d ^(7)^e ^14.3%^f^	6 (18) 33.3%	2 (17) 11.8%	9 (42) 21.4%	6 (7) 85.7%	13 (18) 72.2%	11 (17) 64.7%	30 (42) 71.4%	6 (42) 14.3%
mucinous	1 (3) 33.3%	0 (1) 0%	1 (1) 100%	2 (5) 40%	1 (3) 33.3%	1 (1) 100%	1 (1) 100%	3 (5) 60%	2 (5) 40%
endometroid	3 (5) 60%	0 (1) 0%	0 (1) 0%	3 (7) 42.8%	2 (5) 40%	0 (1) 0%	0 (1) 0%	2 (7) 28.6%	1 (7) 14.3%
clear cell^g^			7(11) 63.6%	7 (11) 63.6%			6 (11) 54.5%	6 (11) 54.5%	5 (11) 45.5%
all types	5 (15) 33.3%	6 (20) 30.0%	10 (30) 33.3%	21(65) 32.3%	9 (15) 60.0%	14 (20) 70.0%	18 (30) 60.0%	41 (65) 63.1%	14 (65) 21.5%

^a^Expression irrespective of the status of the other TACC protein;

^b^Tumor cores expressing both proteins;

^c^Sixty five cores could be graded (1,2,3) and scored for TACC1 and TACC3 expression. Tumors with less than 10% of cells stained were classed as negative (-), greater than 10%, as positive (+). Percentage expression was calculated within each histological subtype.

^d^Number of positive cores in a set;

^e^Total number of cores in a set;

^f^Percentage of positive cores in a set;

^g^Clear cell carcinomas cannot be graded using the World Health Organisation or FIGO systems [13], but are classified as grade 3, as recommended by the NCCN practice guidelines [14].

Next, we examined the association of expression of each TACC protein with specific types of tumor to test whether the distribution of each TACC varied between subtypes (Summarized in Table 1). TACC3 expression was observed in 21.4% (9 of 42) serous papillary, 40% (2 of 5) mucinous, 42.8% (3 of 7) endometroid, and 63.6% (7 of 11) clear cell ovarian tumor cores. A significant difference in TACC3 expression between tumor types was noted, with TACC3 being expressed less frequently in serous papillary tumors than clear cell tumors (Fisher's exact test, p = 0.0113). In contrast, TACC1 was detected in 71.4% (30 of 42) serous papillary, 60% (3 of 5) mucinous, 28.6% (2 of 7) endometroid and 54.5% (6 of 11) clear cell ovarian tumor cores. TACC1 expression was less frequent in endometroid than in serous papillary tumors (Fisher's exact test p = 0.0406). Noticeably, TACC3 compared with TACC1 staining in serous papillary tumors was significantly different (Fisher's exact test, p < 0.0001) (Fig. 2). There was no significant difference between their expression in clear cell, endometroid or mucinous tumor types. However, as it is more difficult to obtain large numbers of these less common types of ovarian cancer, it should be stressed that further analysis will be required to investigate whether relationships between tumor type and grade will hold in a larger sample set.

### Mutation analysis of the TACC3 gene in familial ovarian cancer

The prevalence of structural aberrations in 4p16 noted during ovarian tumorigenesis indicates that this region could contain one or more genes whose normal role is to control the growth and maintenance of the ovarian surface epithelium [3-5]. Thus, loss of one copy of 4p16 in the developing ovarian tumor may result in haploinsufficiency, or unmask a pathogenic mutation in one or more of the genes in this region. With the evidence from the expression analysis presented above, it would appear that a significant number of ovarian cancers lack TACC3 expression. As the TACC3 gene is located within 4p16 [11], we next investigated whether constitutional mutations occur within the TACC3 gene in familial ovarian cancer. Thus, mutation analysis was performed on the TACC3 coding sequence, and the corresponding intron/exon boundaries of this 16 exon gene. Specifically, we used the Transgenomics WAVE dHPLC technology as a pre-screening approach to sample constitutional DNA from ovarian cancer patients and their unaffected relatives from 76 families from the Gilda Radner Familial Ovarian Cancer Registry. These families had previously tested negative for mutations in known ovarian cancer predisposition genes, including BRCA1 and BRCA2, and thus the underlying genetic predisposition in these families is currently unknown [2].

We identified novel sequence variants in a number of the samples, but most of these were due to polymorphisms in the intronic sequence included in the amplified products. However, novel sequence substitutions were also identified in the TACC3 coding sequence (Table 2), many of which resulted in no change at the protein level. Four nucleotide substitutions, not found in the GenBANK or SNP databases, which did alter the protein sequence were identified in both normal and affected sibs and thus also categorized as novel polymorphisms. In most cases, the alterations resulted from C to T base changes in CpG dinucleotides. Intriguingly, we identified a relatively rare insertion/deletion polymorphism in two 12mer amino acid repeats encoded by exon four [11]. The identification of this polymorphism could explain the previously reported variable number of copies of a distinct 24 amino acid repeat found in mouse TACC3 cDNAs from various sources [11,18,21]; although in only one case was the corresponding exon (exon 4) sequenced [18].

**Table 2:** TACC3 sequence changes detected in members of the Gilda Radner registry

Exon	Codon Change	Nucleotide change^a^	Effect^b^	Change in amino acid/protein property	Remarks
3	TCA>TTA	c.278C>T	Ser93Leu	Hydrophilic to hydrophobic	In proband (ovarian cancer), and mother (uterine cancer); not in two unaffected sisters.
4	GAG> AAG	c.427G>A	Glu143Lys	Acidic to basic	Polymorphism
4	AGC>AGT	c.531C>T	Ser177Ser	Silent	Polymorphism
4	c.673_708del AAAGCGGAGACTCCGCACG GAGCCGAGGAAGAATGC		Lys225_Cys236del	Removal of one 12 amino acid repeat	Polymorphism
4	GGC>GGG	c.1086C>G	Gly362Gly	Silent	Polymorphism
4	Ccg>Ctg	c.1250C>T	Pro417Leu	Potential tertiary structure change	Polymorphism
5	GCG>GTG	c.1406C>T	Ala469Val		Polymorphism
6	GGG>GAG	c.1541G>A	Gly514Glu	Small hydrophobic to acidic	Only in affected sibling, not in unaffected sister or daughter.
9	TTC>TTT	c.1809T>C	Phe603Leu	Aromatic to aliphatic	Polymorphism
11	CAC>CAT	c.1998C>T	His666His	Silent	Polymorphism
16		c.2621T>A			3' untranslated region. Polymorphism

^a^: Based upon RefSeq TACC3 nucleotide sequence NM_006342

^b^: Based upon RefSeq TACC3 Protein sequence NP_006333

Significantly, in two unrelated patients from different families, we identified sequence alterations that were not present in unaffected sib(s) in the respective families or unrelated samples. In one case, a heterozygous C to T mutation in exon 6 was detected in the affected sister, but not her unaffected sister or sibling (Table 2). This mutation results in the dramatic change of amino acid 514 from glycine to negatively charged glutamic acid, and was not identified in any other normal or affected individuals from the registry, so far tested. Cross-referencing this sequence to GenBANK revealed only five occurrences of this mutation in cDNAs from the Expressed Sequence Tag database, all of which were derived from tumor tissues (including brain tumors and leukemias). The underlying c.1541G>A nucleotide substitution was detected in the SNP database from a survey of 71 individuals (subdivided into three panels based upon ethnicity), with the minor A allele frequency ranging from 0.312 in the Caucasian North American panel to 0.109 in the African American panel, frequencies that are higher than our detection of this allele in the Gilda Radner registry i.e. in one affected individual in the 76 families tested. In the second family, a heterozygous constitutional mutation was detected in exon 3 that resulted in a serine-leucine change at amino acid 93 in a patient diagnosed with clear cell ovarian carcinoma. This sequence change was not identified in other individuals from the registry, or in the GenBANK and SNP databases. The mother of this patient was previously diagnosed with uterine cancer (although the exact subtype of tumor was not recorded), and was also heterozygous for the same mutation. Two sisters, who are currently disease-free, do not carry the mutation. These data suggest that TACC3 may be a new familial predisposition or modifier locus for gynecological cancer.

## Discussion

TACC1 and TACC3 are expressed in the epithelial components of several tissues in the body, including the mammary gland and the ovary. Based upon inferences from current SAGE data and accumulating genomic analysis, the specific goal of this report was to determine the potential significance of these proteins in the development of ovarian cancer. This has revealed that 78.5% of ovarian tumors lack appreciable expression of TACC1 and/or TACC3. In particular, we have determined that 67.7% (44 of 65) of ovarian tumors lose expression of TACC3. Subdivision of the tumors suggested a difference in the distribution pattern of expression of the two TACC proteins, with TACC3 loss being more common in serous papillary carcinoma compared with clear cell carcinomas, while TACC1 staining was less frequent in endometroid than in serous papillary tumor cores. However, due to the relatively small numbers of tumors in each histological category, particularly the rarer endometroid and mucinous subtypes, firm conclusions about the exact distribution pattern will require analysis of a much larger sample set. Furthermore, we detected constitutional mutations in the TACC3 gene in ovarian cancer patients from the Gilda Radner Familial Ovarian Cancer registry, in the absence of mutations in known predisposition genes.

During growth and progression, tumors can undergo a substantial amount of genomic rearrangement, including translocations, deletions and amplifications. Although many of these changes are random, due to the inherent genomic instability that can occur during tumor cell division, consistent abnormalities in the same or related tumor types can suggest that one or more genes in a particular region may be involved in the pathogenesis of the disease. Several different cancers, and in particular, breast, ovarian, endometrial and cervical cancers exhibit loss of 4p16 [5,22-27], the site of the TACC3 gene [11,10]. Thus, if TACC3 were a significant player in the pathogenesis of cancer, we would expect that a proportion of these tumors could similarly lose expression of TACC3. Indeed, in a survey of 500 resected breast tumors, TACC3 protein was significantly reduced in approximately 50% of the tumors [28]. In contrast, Affymetrix microarray analysis has revealed that levels of TACC3 mRNA increase during the transition of breast cancer from preinvasive ductal carcinoma *in situ *to invasive ductal carcinoma [29], suggesting that TACC3 may impart a proliferative advantage to a subset of breast cancers. A more recent study has indicated that TACC2 and TACC3 are members of a set of 21 proteins that are strong prognostic indicators of clinical outcome in breast cancer [30]. In addition, TACC3 is located within 200 kb of a translocation breakpoint cluster region associated with multiple myeloma, which results in TACC3 upregulation. This suggests that an increase in TACC3 may contribute to the pathogenesis of this B-Cell disorder [11]. A similar dichotomy in the expression pattern of TACC1 has been observed, in that TACC1 can be upregulated or lost in cancer [28,31,32]. This may in part be explained by as yet unidentified tissue specific functions of these proteins or the existence of cancer-associated alternative splice products, as evidenced for TACC1 in gastric cancer [32]. However, currently there is no firm evidence for alternative splicing of TACC3 in normal or cancerous tissues.

Each human TACC gene maps to a region of the genome that is consistently associated with tumorigenesis and progression. Rearrangements of the short arm of chromosome 8 are noted in several different cancers [3,33-35]. Typically, these rearrangements span a significant portion of 8p21-8p11, encompassing the TACC1 locus. Similarly, loss of chromosome 10, in particular the region of chromosome 10q25-26 flanked by the DNA markers D10S221 to D10S216, which encompasses the TACC2 locus, is a frequent occurrence in cancers of different origins (Reviewed in [36]). While this could suggest that the TACC genes may be mutated in a proportion of these cancers, to our knowledge, no report has directly assessed this possibility. With the prevalence of aberrations in the expression of TACC3 in the ovarian tumor arrays, we screened the coding sequence of the TACC3 gene in 76 ovarian cancer families that do not have mutations in known ovarian predisposition loci, such as BRCA1 and BRCA2 [1,2]. This led to the identification of several new coding polymorphisms, which were found in normal and affected individuals in our test subjects. With the relatively late age of onset of ovarian cancer, it remains possible that some of the siblings that have been classified as normal may actually be asymptomatic carriers. Thus, some of these novel polymorphisms may actually represent low penetrance modifiers of ovarian cancer risk, in a similar manner to BARD1 polymorphisms that were subsequently shown to be associated with increased breast cancer risk in the general population [37-39].

In addition, we identified two germline constitutional missense mutations that were specific to the affected patient, and were not found in currently unaffected siblings or unaffected members of the Gilda Radner registry. In addition, the Ser93Leu substitution was not found in normal individuals or cDNAs from the SNP or GenBANK databases. Significantly, the mother of the patient that carried the Ser93Leu substitution was also heterozygous for the same mutation and had previously been treated for a uterine cancer of undetermined type, while two siblings without this mutation remain disease free to date. In the Gilda Radner registry, the risk for uterine cancer is approximately five fold higher than the general population, and this risk increases with the number of first degree relatives diagnosed with ovarian cancer [40]. A similar finding was observed in a separate genealogy-based study [41]. Indeed, a link between ovarian and uterine cancer may not be surprising given the common embryonic origin of the epithelium of the female reproductive tract i.e. the coelomic epithelium [42]. In an independent study, in one BRCA1 linked family, the daughter of a patient diagnosed with ovarian cancer not only had bilateral breast cancer, but also uterine leiomyomata [43,44]. In addition, a germ line missense mutation in the BRCA1 associated protein BARD1 can give rise to independent tumors in the breast, ovary (clear cell carcinoma) and endometrium (also a clear cell carcinoma) [37]. This connection is particularly intriguing considering the recent observation that the *C. elegans *TAC protein interacts directly with the *C. elegans *homologue of BARD1 [45]. Unfortunately, we were unable to obtain additional tumor samples from our patients or material from an additional affected family member for further study. The Gly514Glu mutation, noted in the second family, is also present in five cDNAs derived from cancer cell lines or tumors in the expressed sequence tag (EST) database. Interestingly, the TACC3 sequence cloned by McKeveney et al [21] also contains the Gly514Glu substitution. Once again, this sequence was derived from a leukemia cell line, not from a normal tissue counterpart, suggesting that mutations in TACC3 may also be a feature of other hematological malignancies, in addition to the previously documented aberrant expression observed in multiple myeloma [46]. Intriguingly, two previous studies have reported an increased risk of ovarian cancer in the daughters of mothers with multiple myeloma [47,48], raising the possibility that TACC3 is a candidate gene contributing to the familial association of these two diseases. Further, larger population studies will be required to confirm whether both these changes represent rare polymorphisms, low penetrance modifiers that contribute to ovarian cancer development and/or are direct pathological mutations.

### Potential functional significance of alterations of TACC3 in the ovarian surface epithelium

The TACC genes were first identified as potential oncogenes and tumor suppressors in breast cancer [10,36,49]. However, it is apparent from their proposed functions in cell division, transcriptional and posttranscriptional control, that they have the potential to interact with pathways that are commonly mutated in several different forms of cancer. TACC3 has an evolutionarily conserved interaction with the microtubule associated proteins and mitotic regulators, chTOG [50], and Aurora A kinase, and can be phosphorylated by the latter (Reviewed in [51]). In addition, a genetic interaction between TACC3 and p53 has been suggested, based upon work carried out in mice [52]. Intriguingly, we have recently found that TACC3 associates with a BRCA1-containing complex (Lauffart et al *unpublished*), and Boulton et al [45] have shown that *C. elegans *TAC directly interacts with the *C. elegans *homologue of BARD1. These functional interactions suggest that loss of the TACC proteins may promote tumor progression by increasing aberrations in centrosomal duplication and function, or DNA damage responses [10,53]. In addition, TACC3 has been shown to interact with nuclear localized transcription factors [18,51,54] and histone acetyltransferases [55]. Overexpression of TACC3 in hematopoietic cells results in aberrant localization of FOG1 [54], a regulator of the GATA transcription factors. Intriguingly, similar to TACC3, two members of this latter family, GATA 4 and GATA 6, are either lost or mislocalized in ovarian tumors [20]. Given the potential ability of TACC3 to interact with proteins known to be involved in ovarian tumorigenesis, our data therefore raise the possibility that TACC3 mutation, mislocalization or loss may serve as alternative mechanisms of functional inactivation of GATA4/6 and BRCA1 leading to the dedifferentiation and malignant development of the ovarian surface epithelium.

## Conclusion

This report documents the first recorded analysis of the TACC genes during the development of ovarian cancer. We have now shown that 78.5% of ovarian tumors lack appreciable expression of TACC1 and/or TACC3 proteins, confirming the inferences made from published SAGE analysis. Together with the novel finding that constitutional mutations in the TACC3 gene may be associated with a subset of familial ovarian/gynecological malignancies, this study, therefore, suggests that the TACCs, and TACC3 in particular, are intimately involved in the mechanisms leading to the development of ovarian cancer.

## Competing interests

The author(s) declare that they have no competing interests.

## Authors' contributions

BL analyzed the tumor microarrays, and performed all statistical analysis and data presentation. MMV and JDB performed the immunohistochemical procedures. WAVE dHPLC and mutation analysis of families from the Gilda Radner Familial Ovarian Registry was carried out by RE, DC, and RAD. IHS conceived and designed the project, analyzed tumor microarray data and drafted the complete manuscript.

## Pre-publication history

The pre-publication history for this paper can be accessed here:


